# Population-Level Portal-Based Anxiety and Depression Screening Perspectives in HIV Care Clinicians: Qualitative Study Using the Consolidated Framework for Implementation Research

**DOI:** 10.2196/48935

**Published:** 2024-01-11

**Authors:** Daniela Zimmer, Erin M Staab, Jessica P Ridgway, Jessica Schmitt, Melissa Franco, Scott J Hunter, Darnell Motley, Neda Laiteerapong

**Affiliations:** 1 Section of General Internal Medicine University of Chicago Chicago, IL United States; 2 Western Institutional Review Board- Copernicus Group Princeton, NJ United States

**Keywords:** HIV, patient portal, clinic staff perspectives, depression and anxiety screening, implementation

## Abstract

**Background:**

Depression and anxiety are common among people with HIV and are associated with inadequate viral suppression, disease progression, and increased mortality. However, depression and anxiety are underdiagnosed and undertreated in people with HIV owing to inadequate visit time and personnel availability. Conducting population-level depression and anxiety screening via the patient portal is a promising intervention that has not been studied in HIV care settings.

**Objective:**

We aimed to explore facilitators of and barriers to implementing population-level portal-based depression and anxiety screening for people with HIV.

**Methods:**

We conducted semistructured hour-long qualitative interviews based on the Consolidated Framework for Implementation Research with clinicians at an HIV clinic.

**Results:**

A total of 10 clinicians participated in interviews. In total, 10 facilitators and 7 barriers were identified across 5 Consolidated Framework for Implementation Research domains. Facilitators included advantages of systematic screening outside clinic visits; the expectation that assessment frequency could be tailored to patient needs; evidence from the literature and previous experience in other settings; respect for patient privacy; empowering patients and facilitating communication about mental health; compatibility with clinic culture, workflows, and systems; staff beliefs about the importance of mental health screening and benefits for HIV care; engaging all clinic staff and leveraging their strengths; and clear planning and communication with staff. Barriers included difficulty in ensuring prompt response to suicidal ideation; patient access, experience, and comfort using the portal; limited availability of mental health services; variations in how providers use the electronic health record and communicate with patients; limited capacity to address mental health concerns during HIV visits; staff knowledge and self-efficacy regarding the management of mental health conditions; and the impersonal approach to a sensitive topic.

**Conclusions:**

We proposed 13 strategies for implementing population-level portal-based screening for people with HIV. Before implementation, clinics can conduct local assessments of clinicians and clinic staff; engage clinicians and clinic staff with various roles and expertise to support the implementation; highlight advantages, relevance, and evidence for population-level portal-based mental health screening; make screening frequency adaptable based on patient history and symptoms; use user-centered design methods to refine results that are displayed and communicated in the electronic health record; make screening tools available for patients to use on demand in the portal; and create protocols for positive depression and anxiety screeners, including those indicating imminent risk. During implementation, clinics should communicate with clinicians and clinic staff and provide training on protocols; provide technical support and demonstrations for patients on how to use the portal; use multiple screening methods for broad reach; use patient-centered communication in portal messages; provide clinical decision support tools, training, and mentorship to help clinicians manage mental health concerns; and implement integrated behavioral health and increase mental health referral partnerships.

## Introduction

### Barriers to Depression and Anxiety Diagnosis

Depression and anxiety are common mental health conditions among people with HIV, with a prevalence of 20% to 45% [1-10]. People with HIV experiencing symptoms of depression or anxiety are more likely to miss appointments and have lower medication adherence, higher HIV viral loads, and higher mortality rates than those without depression or anxiety [1,2,5,10,11]. However, depression and anxiety are often underdiagnosed and undertreated in people with HIV, particularly among African Americans and Hispanics, because of the perceived stigma of mental health disorders, racial discrimination, HIV-related discrimination, and medical mistrust [1,3,5,12,13]. In the HIV Cost and Service Utilization survey of people with HIV identified as experiencing depression, only 45% had a formal depression diagnosis in their medical chart [14].

For people living with chronic conditions, such as HIV, specialty care clinics often serve as their primary source of health care [3,4]. Given the frequency of visits people with HIV have with their HIV care team, establishing mental health screening in HIV clinics is a key opportunity to address depression and anxiety underdiagnosis in people with HIV [3,4]. Patients and physicians have noted that depression screening in clinics is helpful in identifying, assessing, and treating depression [3]. However, competing demands and priorities during appointments, a lack of staff to complete assessments, and a shortage of resources to offer patients after diagnosis discourage clinicians from screening and treating depression [1,3,6,7,9,15,16].

### Novel Mental Health Screening

A novel strategy to increase depression and anxiety screening in people with HIV is to perform screening at the population level using the patient portal. In recent years, health care systems have increased the adoption of electronic patient portals, and patients have increasingly used portals to facilitate their health care [8,9,17-20]. Studies in primary care settings have found that depression screening rates increased significantly when clinics adopted portal-based screening [3,7,18]. Notably, a population-level portal-based depression screening intervention, in which patients were invited to complete a depression screener regardless of having a scheduled appointment, also increased depression screening and diagnosis rates [21]. This population-level portal-based approach identified more patients with moderate to severe symptoms than screening during clinic appointments [22]. Moreover, portal-based screening increases the likelihood of discussing depression diagnosis and treatment during an appointment [7,19,20,22].

Population-level portal-based screening has been shown to be promising in primary care settings but has not been examined in HIV care settings. Guided by the Consolidated Framework for Implementation Research (CFIR), we conducted and analyzed qualitative interviews with clinicians at an urban HIV clinic [23]. This study explored clinicians’ perspectives on facilitators of and barriers to implementing population-level portal-based depression and anxiety screening for people with HIV. The objective of this formative study was to use the identified facilitators and barriers to develop implementation recommendations for HIV clinics.

## Methods

### Study Design

We completed a qualitative study to inform the design and implementation of population-level portal-based depression and anxiety screening at an HIV clinic. This paper reports the results of interviews conducted with clinicians and clinical staff.

### Study Setting

The study was conducted in the Ryan White HIV Care Clinic at an academic medical center on the South Side of Chicago, the main provider of HIV care services for Chicago South Side residents. The South Side of Chicago is one of the communities most impacted by the HIV epidemic in the United States [24]. The clinic provides care for >630 people with HIV, most of whom are African Americans and publicly insured. Currently, staffed with 15 physicians, 6 fellows, a nurse practitioner, a licensed practical nurse, 2 pharmacists, and 2 licensed social workers, the clinic also provides mental health services.

In November 2020, the HIV clinic adopted a protocol for conducting depression and anxiety assessments during in-person clinic visits. Medical assistants were asked to complete the 2-item Patient Health Questionnaire (PHQ) and the 2-item Generalized Anxiety Disorder (GAD) scale with patients due for annual screening, as indicated by health maintenance topics and best practice advisories in the electronic health record (EHR) [25,26]. Scores of ≥3 were reflexed into the PHQ-9 and GAD-7, respectively. Medical assistants were also asked to complete the PHQ-9 and GAD-7 with patients with a history of depression or anxiety, respectively, who were due for ongoing symptom monitoring or surveillance. Physicians and advanced practice nurses were alerted via a critical, noninterruptive best practice advisory to scores of ≥3.

Concurrent with this study, a population-level portal-based depression screening intervention was tested in the primary care clinic at the academic medical center. Patients were invited to complete depression screening using the patient portal regardless of having a scheduled appointment [21]. The clinic saw an increase in screening and identification of depression [21]. These advances in screening in the primary care clinic at the institution motivated us to gauge the interest in and feasibility of integrating population-level portal-based screening in the HIV clinic.

### Study Participants

All clinicians at the HIV clinic were eligible for study enrollment, including physicians, advanced practice nurses, pharmacists, nurses, and social workers. HIV clinicians were informed about the study at a clinic meeting and by email, and if interested in participating, they were instructed to contact the study project manager. The participants verbally consented before each interview.

### Data Collection

Semistructured interviews were conducted one-on-one with each participant from January to April 2021, after the newly adopted in-clinic screening protocol was implemented. Demographic information was collected via electronic surveys in the REDCap (Research Electronic Data Capture; Vanderbilt University). Interviews were conducted over Zoom (Zoom Video Communications) and lasted for approximately 60 minutes. The interview questions were created by the research team using the CFIR interview guide tool for all 5 CFIR domains. The full interview guide is available in Multimedia Appendix 1. The following are example questions by domain:

- Innovation characteristics: “Do you think assessing anxiety and depression using the patient portal will be effective? Why or why not?”
- Inner setting: “What is the general level of receptivity in the clinic to using the patient portal?”
- Outer setting: “Do you think measuring anxiety and depression using the patient portal will meet the needs of the patients served by your clinic? Why or why not?”
- Characteristics of individuals: “How do you feel about this method of assessing anxiety and depression in the HIV care clinic? Anticipation? Stress? Enthusiasm? Why?”
- Process: “Who are other key influential individuals to get on board with assessing anxiety and depressive symptoms using the portal?”

### Data Analysis

Descriptive analysis was used to summarize the characteristics of the participants interviewed. Initially, the original CFIR domains and constructs from the codebook were used for our interview analysis. During the initial coding phase, research team members identified additional themes and subthemes to be added to the interview analysis. Once consensus was achieved on the codebook for our analysis, 2 independent coders analyzed each interview transcript, and coding discrepancies were discussed until a consensus was reached. Analysis of coded transcripts was performed in the web-based software Dedoose (version 9.0.17; SocioCultural Research Consultants, LLC). A total of 2 research team members independently reviewed the coded excerpts to find common themes within each domain, identified each as a facilitator or a barrier, and discussed them to consensus. On the basis of these facilitators and barriers, the 2 research team members proposed implementation strategies and presented these strategies to the entire study team for validation and refinement.

### Ethical Considerations

The study was reviewed and approved by the University of Chicago Biological Sciences Division Institutional Review Board (20-1313). The research team obtained oral consent from participants before the beginning of the interviews. Interview audio was recorded, and transcripts were deidentified before qualitative coding. The data were accessible to the research team only. Participants were given a US $40 e-gift card for interview completion.

## Results

### Participant Characteristics

Interviews with HIV clinicians continued until the team agreed that data saturation was met, as indicated by the lack of new themes emerging in the interviews. In total, 10 HIV clinicians completed the interviews. The participants ranged in age from 31 to 64 years. Most participants were identified as White (8/10, 80%) or male (6/10, 60%). As shown in Table 1, 70% (7/10) worked as physicians at the HIV clinic, and the remaining staff included a social worker (1/10, 10%), a nurse (1/10, 10%), and a pharmacist (1/10, 10%).

**Table 1:** Demographic information of interview participants.

Characteristics			Participants (N=10), n (%)
**Age (y)**			
	30-39	5 (50)	
	40-49	2 (20)	
	50-59	2 (20)	
	60-69	1 (10)	
**Sex**			
	Male	6 (60)	
	Female	4 (40)	
**Race**			
	Asian	1 (10)	
	Black or African American	1 (10)	
	White	8 (80)	
**Clinical role**			
	Physician	7 (70)	
	Social worker	1 (10)	
	Nurse	1 (10)	
	Pharmacist	1 (10)	
**Caring for people with HIV (y)**			
	1-5	3 (30)	
	6-10	2 (20)	
	11-15	2 (20)	
	≥15	3 (30)	
**Clinical experience (y)**			
	1-5	5 (50)	
	6-10	2 (20)	
	≥15	3 (30)	

### Current Depression and Anxiety Screening Practice

When asked about their current mental health screening practices, most participants mentioned informally screening patients by asking how they were feeling or if the patient was experiencing any thoughts of self-harm or suicidal ideation. As 1 participant said, “Within the review of systems [during patient intake], I often will ask if any depression and anxiety-type symptoms [were experienced] recently, but there is no standard way I approach every patient” (Participant 6, physician). Participants reported that patients expressing depression or anxiety would typically be screened with the PHQ and the GAD questionnaire. A few participants spoke of the newly implemented in-clinic mental health screening procedure put in effect before the interviews were conducted. However, they stated that the screening protocol was not regularly followed during the clinic visits.

Similarly, the participants mentioned that initiating mental health services relied on patients requesting services or bringing up suicidal ideation or self-harm. The clinic relies on HIV-trained clinical social workers to connect patients with mental health resources based on the individual’s insurance. One participant explained, “I’ll have our social worker call them and set them up with a resource and have her—obviously she’s more trained in that than I am, I believe—and have her assess them and provide appropriate resources” (Participant 9, physician). Before the COVID-19 pandemic, patients were regularly introduced to social workers during in-clinic appointments. The participants emphasized that these in-person interactions were valuable, as they built trust between the patient, the social worker, and the physicians to increase intervention uptake.

### Perceptions of Population-Level Portal-Based Screening

#### Overview

Facilitators of and barriers to population-level portal-based depression and anxiety screening were identified within the 5 CFIR domains (Tables 2 and 3).

**Table 2:** Facilitators to population-level portal-based depression and anxiety screening from qualitative interviews with HIV clinicians.

Domain and facilitators		Facilitator quotes
**Innovation characteristics**		
	Advantages of systematic screening outside clinic visits	“I like the idea that it feels like it would make it a little less stressful on the clinic visit because it’s done independently of that. And then it might come up during the visit, but at least the initial screening and questions doesn’t get added to that clinic workflow. So from that standpoint, it’s a little bit relieving. Because we do want to do this, but you don’t want to have anyone having too much work or too many things on their plate. So from that standpoint, it feels like a good method to go about it.” (Participant 2, physician)
	Expectation that assessment frequency could be tailored to patient needs	“I think if we did it this way, then we would have the information at the beginning of a visit, and could then walk into the visit knowing this. And maybe even have some additional background from our social worker, if they’ve reached out to them in the meantime, between the time they filled this out and got these results, and then the time we see it for an appointment.” (Participant 8, physician)
**Outer setting**		
	Greater respect for patient privacy	“I kind of think that’s where you get the most honest answers, in the patient’s environment. In clinic, the patient’s mental status is, I’m ready for clinic. So they have that person put on, their clinic person. And unless something is like really, really outstanding, they’re not forthcoming with their information, right?” (Participant 4, nurse)
	Normalizing mental health screening	“...what I’m thinking is I really liked the way it is being approached as making it a routine part of HIV [care]...just destigmatizing and routinizing those questions for people. I think once that becomes routine as part of your, whatever, yearly check-in, I think that’s helpful.” (Participant 3, social worker)
**Inner** **setting**		
	Compatible with clinic culture, workflows, and systems	“Overall positive feelings towards it. I like the idea that it feels like it would make it a little less stressful on the clinic visit because it’s done independently of that. And then it might come up during the visit, but at least the initial screening and questions doesn’t get added to that clinic workflow. So from that standpoint, it’s a little bit relieving. Because we do want to do this, but you don’t want to have anyone having too much work or too many things on their plate.” (Participant 2, physician)
	Protocol for addressing positive screening results	“Would be nice to have a pathway that’s somewhat predetermined. So, it’s like, ‘Okay, we identified this patient has this. We’re not sure if they’re going to be able to see a mental health provider because it might take 2 months to get in.... But in the meantime, this is the plan. This is our protocol for what we should do. These are first-line medications. This is the plan from our social work standpoint of how we’re going to follow up with them.’ So, things like that. That would make it easier once we do identify the need to take some of the guesswork out of what the next steps are.” (Participant 8, physician)
**Characteristics of individuals**		
	Participant beliefs about the importance of mental health screening and benefits HIV care	“Some of it could be a little bit more work within the appointment if you’re then talking about some of these issues and how they affect their other medical care, but I think it would be time likely worth spent and gratifying, and probably maybe more time spent in the front would help decrease time later needing to if it were something that could be addressed and then would improve compliance, that would be very meaningful and worth discussion.” (Participant 6, physician)
	Participant interest in evidence-based practices and desire to learn from prior implementation	“I think there’s a strong need to do it in general.... I haven’t read all the literature on it.... Most likely this needs to happen. We need to screen people. And then the question is just, ‘What’s the best way to screen?’ And looking at everything and talking through the pros and cons, it feels like this would probably be a good way to do it.” (Participant 2, physician)
**Process**		
	Team-based approach that leverages strengths of all clinic staff	“Our patient population is a bit delicate, which is why we have different levels to our team approach, because what patients wouldn’t share with their doctor they will share with me, because they easily identify with me. So they accept it on an extended family member kind of like basis. So their level of trust is greater. And we use that. It’s very effective.” (Participant 4, nurse)
	Clear planning and communication with staff	“In general having something that’s standardized is good. Having something that doesn’t totally disrupt the workflow in clinic. So using the patient portal is excellent. And having a really clear plan for what the follow-up is for the patient. I think those are the really important things. And if those are well communicated to the clinic, to the section beforehand.... We have our Monday meetings at noon, something like that.... So that way everyone’s comfortable. I would be comfortable going forward with something like this, but making sure that everyone’s on the same page.” (Participant 2, physician)
	System that empowers patients to communicate about their mental health	“But most of our patient population is a very secretive population. So I believe being able to have something on their own terms...[The social workers could] be like, ‘Hey, if you ever feel A, B, and C.... Hey just answer these questions. I get an alert and I will respond or someone will respond in a reasonable timeframe.’ Yeah. So if patients have the information that you can use MyChart to let us know if something is going on, I think that would be more successful than just screening patients as they check-in in clinic.” (Participant 4, nurse)

**Table 3:** Barriers to population-level portal-based depression and anxiety screening from qualitative interviews with HIV clinicians.

Domain and barriers		Barrier quotes
**Innovation characteristics**		
	Difficulty of ensuring prompt response to those in imminent risk to themselves or others	“I’d want to make sure that with this screener that we’re assessing for suicidal or homicidal ideation and that somehow that gets like flagged to be address immediately because when you’re in the clinical setting, you can address it immediately. But over the portal, I worry that it might just like sit there, and then what happens if someone is actively suicidal and they fill this out and nobody addresses it.” (Participant 8, physician)
**Outer setting**		
	Limited patient access, experience, and comfort using the portal	“And the reality, with my patient population, there has been issues with just accessing MyChart for a variety of patient problems, if you will. Lack of technical skills, lack of just having no laptop or any way to do that, or just feel comfortable with that kind of thing.” (Participant 5, physician)
	Limited availability of mental health services	“Our barrier is the resource pool that we have to select from...we’re extremely limited...if someone is not a threat to themselves or someone else, however they’re battling their issues that are too much for them to really handle, where do we refer our patients to? And the destinations are booked out. And I personally believe that time is a factor when we’re dealing with depression and anxiety.” (Participant 4, nurse)
**Inner setting**		
	Clinician variation in the use of electronic health records	“I think a lot of the physicians I work with don’t even check their in-basket, answer My Chart messages...I mean, I’ve been using it and I do like it.... But I think a lot of the people I work with.... They trained in a different time, none of this was around then. A lot of them give out their cell phone numbers to their patients and that’s how they end up communicating.” (Participant 10, physician)
	Limited capacity to address mental health concerns during HIV visits	“We have a list of our own priorities that we need to address every visit. I think sometimes I’m like, ‘Well, they have a primary care physician. That’s the appropriate person that should assess and counsel, and hopefully they’re doing that.’ I kind of rely on that. And probably we’re often not as good at realizing when someone is in some kind of mental health distress. We see someone and they might seem like they’re doing okay and I’m like, ‘I don’t need to ask them how their mental health is.’ But obviously under the surface could be a very different story.” (Participant 9, physician)
**Characteristics of individuals**		
	Participant concerns about limited knowledge about mental health treatments	“I think that a lot of the actionable information or the action that I’ll most likely take will eventually fall on the [infectious disease] clinic social worker [based] on my previous behavior.... I’m not likely, to be honest, to start any medication. I just don’t feel well-versed enough or practiced enough to really prescribe pharmacologic interventions. So usually the interventions I would take are to refer them to their provider or have our social worker kind of provide resources in some way. I don’t feel equipped to provide nonpharmacologic interventions related to anxiety/depression or pharmacologic.” (Participant 9, physician)
**Process**		
	Impersonal approach to sensitive topic of mental health	“As you know, it takes patient buy-in to be able to feel like you’re not...exploiting people almost.... You’re getting into mental health and that sometimes can be a touchy subject to do in an impersonal manner, I would imagine, through something like an email or a text or MyChart.... I can envision certain patients not really warming up to it, just because it is impersonal and you’re just filling out.... I’m sure the patients, if they have the idea that this is totally for their upcoming visit and we just want to make sure we’re being complete and we want to take care of you, if there’s any concerns in the realm of depression/anxiety, we’d like to be able to address them appropriately.” (Participant 5, physician)

#### CFIR Domain 1: Innovative Characteristics

Codes within the innovation characteristics domain focused on the attributes of population-level portal-based screening. The participants spoke about its relative advantage, adaptability, complexity, evidence base, and design quality.

##### Facilitator: Advantages of Systematic Screening Outside Clinic Visits

The participants thought that population-level portal-based mental health screening would help make screening more consistent without imposing additional work or disrupting the clinic workflow. With screening completed ahead of time via the patient portal, the participants felt that they would be better prepared to address these concerns during the visit.

##### Facilitator: Expectation That Assessment Frequency Could be Tailored to Patient Needs

Participants generally thought that sending assessments via the portal every 6 months or once a year would be ideal; however, they mentioned that they would defer to the evidence on screening frequency best practices. The ability to send patients mental health screeners at a custom interval appealed to participants as it would keep them aware of the mental health concerns that arose. The participants also expressed interest in tailoring the screening frequency based on symptom severity.

##### Barrier: Difficulty Ensuring Prompt Response to Those in Imminent Risk to Themselves or Others

A common concern raised during the interviews was the complexity of responding to patients who indicated suicidal risk, self-harm, or homicidal ideation on the screener. Participants were worried that patients with immediate mental health needs would not receive timely interventions if screening were performed via the portal.

#### CFIR Domain 2: Outer Setting

Codes within the outer setting domain focused on external factors that might affect the implementation of population-level portal-based screening, particularly the needs and resources of the patient population served by the clinic.

##### Facilitator: Greater Respect for Patient Privacy

Participants spoke about how patients seen at the HIV clinic value privacy. They thought that using the portal might increase screening uptake and encourage honest responses by allowing patients the flexibility to complete screening in environments where they are most comfortable.

##### Facilitator: Normalizing Mental Health Screening

Participants recognized the stigma associated with mental illness for some patients. Implementing a routine depression and anxiety screening process was seen as an approach to destigmatize mental health assessments. In addition, participants believed that consistent depression and anxiety screening would frame mental health as part of patients’ general health care, compared with the sporadic mental health assessments in current practice.

##### Barrier: Limited Patient Access, Experience, and Comfort Using the Portal

The participants did not know if patients were familiar enough with the portal to complete the assessments electronically. The participants reported that several of their patients did not know how to access their laboratory work via the portal. Therefore, they were not confident that the patients could complete screeners via the portal without assistance or training. The participants were also concerned about usability issues regarding the small text and reading levels associated with using the portal.

##### Barrier: Limited Availability of Mental Health Services

Participants emphasized that accessible mental health referral pathways and resources were needed before the clinic could implement population-level portal-based screening. Otherwise, patients would be diagnosed without the proper resources to be treated. Although the clinic has existing partnerships with external mental health facilities, waitlists were long. Furthermore, transportation, insurance, and cost barriers limited patients’ access to mental health treatments. In addition, concerns about the capacity of the current referral network to handle an influx of newly diagnosed patients were expressed by participants.

#### CFIR Domain 3: Inner Setting

Codes within the inner setting domain focused on the clinic’s characteristics and readiness to implement population-level portal-based screening. Participants spoke about compatibility, available resources, access to knowledge and information, networks and communication, and culture.

##### Facilitator: Compatibility With Clinic Culture, Workflows, and Systems

Participants strongly expressed interest in implementing population-level portal-based screening for depression and anxiety through the portal and thought their colleagues would also be receptive. Participants stated that this would help create an open relationship with patients while prioritizing clinical values to provide holistic care to their patients. Participants said that using the portal for depression and anxiety screening would provide crucial information on patients’ mental health status without adding significant stress to clinical workflows.

##### Facilitator: Protocol for Addressing Positive Screening Results

Participants wanted a systematic process to manage patients who screened positive to avoid delays in connecting patients to resources. Specifically, participants expressed a desire for detailed guidance on available resources, referral pathways, and a follow-up plan for symptomatic patients. A few participants were interested in additional training or decision support tools to help them interpret screening results, connect patients to resources, and prescribe first-line medications.

##### Barrier: Clinician Variation in the Use of EHRs

Participants expressed concern that screening results in the EHR might be overlooked because clinicians did not always check their electronic in-baskets reliably because of variations in their proficiency and comfort with the EHR.

##### Barrier: Limited Capacity to Address Mental Health Concerns During HIV Visits

Participants raised concerns about having adequate personnel, time, and expertise to manage depression and anxiety. They reported limited time during appointments to address their patients’ health issues and social needs, and there may not be enough time to address depression and anxiety management.

#### CFIR Domain 4: Characteristics of Individuals

Codes within the individual characteristics domain focused on the participants’ knowledge, beliefs, and self-efficacy.

##### Facilitator: Participant Beliefs That Mental Health Screening Is Important and Benefits HIV Care

Most participants agreed that mental health screening was essential and valuable. They saw population-level portal-based screening as an opportunity to learn more about their patients and to address concerns that might not otherwise arise during appointments. Participants recognized the effects of mental health problems on HIV outcomes and were hopeful that addressing depression and anxiety would improve engagement with care and general health.

##### Facilitator: Participant Interest in Evidence-Based Practices and Desire to Learn From Prior Implementations

Participants indicated their willingness to abide by evidence-based mental health screening and management recommendations. They expressed a desire to learn more about how population-level portal-based depression and anxiety screening had been implemented in the primary care clinic so that the lessons learned could be applied to the HIV clinic.

##### Barrier: Participant Concerns About Limited Knowledge About Mental Health Treatments

Some participants were hesitant to implement population-level portal-based mental health screening because they thought that they lacked adequate expertise in mental health treatment and navigating mental health resources.

#### CFIR Domain 5: Process

Codes within the process domain focused on planning the intervention and engaging clinicians and patients.

##### Facilitator: Team-Based Approach That Leverages the Strengths of All Clinicians

Participants believed that a team approach would be crucial for successfully implementing population-level portal-based screening. Social workers were identified as key team members to provide knowledge on available resources and support connecting patients to care. Participants also indicated that engaging clinicians with strong relationships with patients would help lower patient hesitancy to engage with the portal.

##### Facilitator: Clear Planning and Communication With Staff

Participants emphasized the importance of having a standardized protocol that included details on which staff member was responsible for each step, especially in response to positive results, and training for all clinic personnel on this protocol before implementation. Participants highlighted the need for clear communication throughout the intervention’s preimplementation, implementation, and sustainability phases. They advised monitoring the intervention logistics and collecting iterative feedback from staff and patients throughout the intervention rollout.

##### Facilitator: A System That Empowers Patients to Communicate About Their Mental Health

Participants thought that population-level portal-based mental health screening could prompt patients to discuss their mental health with their care team. The participants believed that providing patients with the flexibility to complete screening assessments at their convenience and through their preferred screening method would empower them to inform their care team about their symptoms. Some suggested that screeners should always be readily available in the portal so patients could report their mental health symptoms as they feel them.

##### Barrier: Impersonal Approach to the Sensitive Topic of Mental Health

Participants expressed concerns that portal-based screening might be impersonal and that unexpected messages about mental health might seem invasive or cause anxiety in some patients. The participants emphasized that clear and patient-centered conversations would need to occur to explain the purpose of mental health screening. Otherwise, the participants feared that patients who did not understand the purpose or context of mental health screening would be unlikely to respond. The participants believed that if patients were informed about how these assessments pertained to their general health, they would be more likely to complete the screeners.

## Discussion

### Principal Findings

This qualitative study explored facilitators of and barriers to implementing population-level portal-based depression and anxiety screening in an HIV clinic. A total of 10 facilitators and 7 barriers were identified across 5 CFIR domains. Facilitators included the following: advantages of systematic screening outside clinic visits; the expectation that assessment frequency could be tailored to patient needs; evidence from the literature and previous experience in other settings; respect for patient privacy; empowering patients and facilitating communication about mental health; compatibility with clinic culture, workflows, and systems; staff beliefs about the importance of mental health screening and benefits for HIV care; engaging all clinic staff and leveraging their strengths; and clear planning and communication with staff. Barriers included difficulty in ensuring prompt response to suicidal ideation; patient access, experience, and comfort using the portal; limited availability of mental health services; variations in how providers use the EHR and communicate with patients; limited capacity to address mental health concerns during HIV visits; staff knowledge and self-efficacy regarding the management of mental health conditions; and the impersonal approach to a sensitive topic. Several barriers mentioned by participants, such as limited appointment times and limited access to mental health resources after diagnosis, are common challenges cited in similar implementation efforts [2,3,6,7,25,27].

Findings from our analysis have been used to compile a list of proposed implementation strategies to help integrate population-level portal-based depression and anxiety screening into practice within the HIV clinic setting.

### Clinician-Focused Implementation Strategies

#### Strategy 1: Conduct a Local Assessment of Clinicians and Clinic Staff

Clinicians and clinic staff are essential to successfully implementing population-level portal-based depression and anxiety screening in the HIV clinic. To increase the feasibility and sustainability of the intervention, clinicians and clinic staff should be asked how the intervention would fit with their beliefs and values, the clinic culture, and its current clinical workflows. Clinicians’ and clinic staff’s thoughts should be incorporated into the implementation plan to assist in intervention compatibility and uptake.

#### Strategy 2: Engage Clinicians and Clinic Staff With Various Roles and Expertise to Support Implementation

The success of the intervention depends on clinicians’ engagement through the implementation process to inform the intervention using clinicians and clinic staff’s strengths. Clinicians and clinic staff in various roles may have different perspectives and ideas on implementing the intervention. Therefore, diversifying the staff perspective may provide crucial implementation strategies that might not be known by only interviewing clinicians.

#### Strategy 3: Highlight Advantages, Relevance, and Evidence for Population-Level Portal-Based Mental Health Screening

Before implementing population-level portal-based mental health screening, the advantages of depression and anxiety screening must be communicated and emphasized to all clinicians. These benefits should highlight how timely mental health discussions between patients and clinicians make efficient use of the limited appointment time. Information on relevant evidence and current clinical screening guidelines should also be provided to garner clinic support. Describing barriers encountered and lessons learned in other practices that have implemented similar interventions could ease concerns about implementation challenges.

#### Strategy 4: Communicate With Clinicians and Clinic Staff Throughout Implementation and Provide Training on Protocols

Training and involving clinicians throughout the rollout of the intervention will facilitate iterative feedback to troubleshoot any challenges that arise and help aid clinicians and clinic staff uptake. As clinicians and clinic staff tend to have established relationships with their patients, receiving their and their patients’ concerns will aid clinicians, clinic staff, and patient engagement throughout the intervention rollout.

### Patient-Focused Implementation Strategies

#### Strategy 5: Provide Technical Support and Demonstrations for Patients on How to Use the Portal

Providing technical support and conducting training on using the portal might increase intervention uptake among patients. Demonstrations could decrease technology-related barriers and encourage patients to use the portal to complete assessments.

#### Strategy 6: Use Multiple Screening Methods for Broad Reach

Multiple screening approaches might be needed to reach all patients attending the clinic. For example, options could include completing depression and anxiety screening in the waiting room before an appointment, over the phone, or during an in-person appointment (eg, during intake before the clinician enters the room). Providing additional screening options for patients who are not technologically proficient or have limited access to technology may increase patient uptake of depression and anxiety screening.

#### Strategy 7: Use Patient-Centered Communication in Portal Messages

Patient-centered messages emphasizing privacy and framing mental health screening as part of routine care can provide a context for portal-based screeners and decrease patient hesitancy to answer questions about the potentially sensitive and stigmatized topic of mental health. Using the patient portal to send patient-centered messages will also allow patients to ask questions about population-level patient-based screening and address concerns.

### IT-Focused Implementation Strategies

#### Strategy 8: Make Screening Frequency Adaptable Based on Patient History and Symptoms

Adaptability of screening frequency and leveraging the staff-patient relationship may improve intervention uptake. Clinicians could adjust the frequency of depression and anxiety screenings based on their relationship with the patient. By allowing staff to adjust the screening frequency, the clinic can check in on patients experiencing uncontrolled depression and anxiety symptoms. Likewise, the staff can lengthen the screening intervals when the patient is in remission for depression and anxiety. This adaptability will signal to patients that the clinic is prioritizing the patient’s health needs.

#### Strategy 9: Use User-Centered Design Methods to Refine How Results Are Displayed and Communicated in the EHR

When designing how portal-based results will be stored and displayed in the EHR, clinicians and clinic staff need to be engaged to ensure the utility of the screening information. Using a user-centered design with these essential stakeholders could increase the likelihood that portal-based depression and anxiety screening will be used in practice.

#### Strategy 10: Make Screening Tools Available for Patients to Use on Demand in the Portal

On-demand assessments would support patient autonomy and allow patients to signal when they are experiencing depression and anxiety symptoms. This patient-centered approach could enhance the clinic’s capacity to treat patients when needed. This differs from the traditional annual one-time screening, which aims to identify depression and anxiety in asymptomatic patients. Traditional screening may increase the demand for services and reduce the clinic’s ability to provide timely and appropriate care for symptomatic patients. Moreover, prioritizing on-demand assessments outside of appointment times could facilitate outreach between appointments and reduce the time to treatment.

### Clinic-Focused Implementation Strategies

#### Strategy 11: Create Protocols for Positive Depression and Anxiety Screening Results, Including Those Indicating Imminent Risk

Establishing a standardized protocol for patients who are symptomatic of depression or anxiety may ease concerns about managing patients who are at imminent risk to themselves or others. For example, the protocol can describe who will contact the patient after the clinic has received a positive PHQ or GAD and how to assist patients in crisis. This will ease clinicians’ concerns about screening patients for depression and anxiety via the portal.

#### Strategy 12: Provide Clinical Decision Support Tools, Training, and Mentorship to Help Clinicians Manage Mental Health Concerns

Providing evidence-based information on treatment or referral strategies through decision support tools, ongoing training, and clinician mentorship for managing mental health treatment would support clinicians’ confidence and ability to manage symptomatic mental health concerns in their patients. Through shared collaborations with mental health specialists and community mental health services, clinicians will be equipped to manage a potential influx of symptomatic patients via the portal.

#### Strategy 13: Implement Integrated Behavioral Health and Increase Mental Health Referral Partnerships

In concurrence with strategy 12, the clinic will need to invest and establish partnerships with local mental health sites to support the clinic’s capacity to treat newly diagnosed patients. Expanding the clinic’s referral network would create a safety net that the clinic can leverage to refer patients. This would prevent long wait times for treatment after a depression or anxiety diagnosis. By creating a behavioral health referral network, HIV clinicians can provide trusted resources to expand the clinic’s internal infrastructure, facilitate warm handoffs with community partners, and continue to support patient care.

### Limitations

The study was conducted at a single academic HIV clinic. Therefore, the results of this qualitative analysis may not be generalizable to other HIV clinics with different patient populations, staffing needs, available resources, and portal uptake. The implementation strategies are merely recommendations from a single HIV clinic and may need to be adapted to fit the implementation setting. At the time of the interviews, clinic staff did not have experience with population-level portal-based mental health screening; therefore, their perspectives were based on how they perceived the intervention would be for patients and themselves once implemented. Although our study included perspectives from clinicians in various clinical roles, most interviewees were physicians, limiting available insight. Gaining patient perspectives through patient-focused interviews would provide further insight into facilitators, barriers, and intervention implementation strategies.

### Conclusions

Our study provides information on clinicians’ views on population-level portal-based mental health screening within the HIV clinic setting. Participating clinicians expressed concerns about the accessibility of prompt mental health resources, patients’ perceptions of mental health screening, variation in clinician use of Epic (Epic Systems, Verona Wi), and limited clinician training on mental health management. Nevertheless, clinicians were interested in establishing population-level portal-based screening at the HIV clinic and were amenable to creating protocols for addressing positive mental health screening, to participating in training about available mental health resources and best practices, and to feeling it was compatible with the clinic. Others may build upon this work by exploring and identifying additional facilitators, barriers, and implementation strategies that were not found in our analysis.

## Appendix

Multimedia Appendix 1 Interview guide.

## Abbreviations

CFIR: Consolidated Framework for Implementation Research
EHR: electronic health record
GAD: Generalized Anxiety Disorder
PHQ: Patient Health Questionnaire
REDCap: Research Electronic Data Capture
