# Supplementary material for: Population-Level Portal-Based Anxiety and Depression Screening Perspectives in HIV Care Clinicians: Qualitative Study Using the Consolidated Framework for Implementation Research
Source: JMIR Form Res. 2024 Jan 11;8:e48935. doi: 10.2196/48935 (PMC10811578; doi:10.2196/48935)
Supplement: Multimedia Appendix 1 [file formative_v8i1e48935_app1.docx]

**Appendix:**

**Interview Guide**

Current practice

1. How do you assess anxiety and depressive symptoms in your clinical care?
2. How do you manage anxiety and depressive symptoms in your clinical care?

Screening in HIV clinic

1. What are your thoughts about a clinic-wide strategy to ask patients about anxiety or depression during HIV care appointments?
2. As part of a clinic-wide strategy, who should be asking these questions about anxiety or depression during HIV care appointments?
3. What kinds of changes need to be made to make a clinic-wide strategy for assessing anxiety and depressive symptoms work effectively in your clinic? For the people we serve in this clinic?

Screening via portal

We are designing a system to screen and monitor anxiety and depression for PLWH using the patient portal (MyChart). We have been able to develop a similar workflow in the General Internal Medicine Clinic. [Show figure and explain]. In this workflow, patients receive an invitation to complete assessments via the portal. This process is automated by IT. The patient then logs in to their portal account and completes the assessment. These results are automatically sent to their provider and stored to the patient’s medical record. A social work reviews the cases of patients who screened positive via the portal and provides follow-up care as needed.


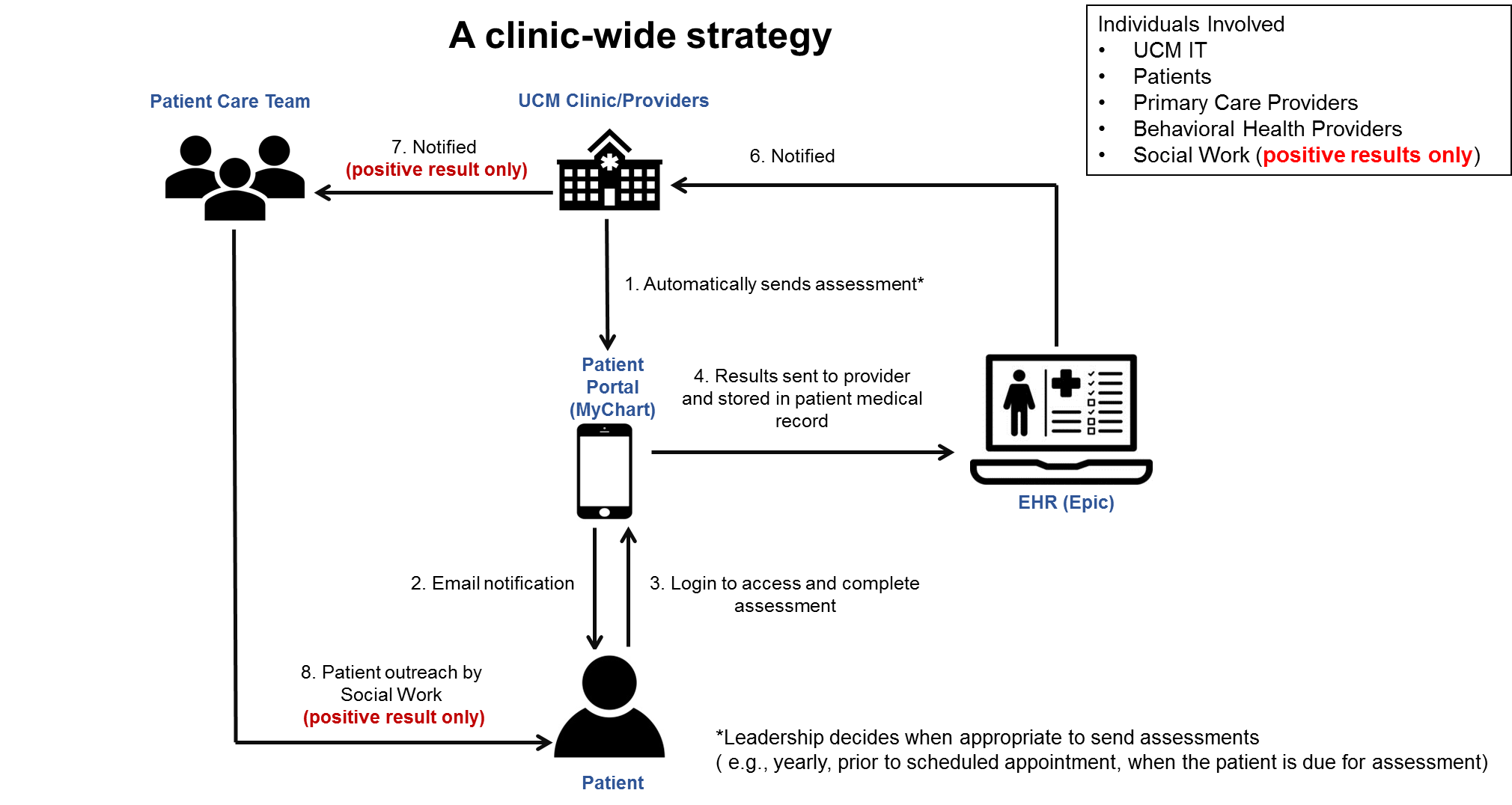


1. What is the general level of receptivity in the clinic to using the patient portal?
2. What do you think about an automated system for assessing anxiety and depression using the patient portal?
3. How do you feel about this method of assessing anxiety and depression in the HIV care clinic? Anticipation? Stress? Enthusiasm? Why?
4. Is there is strong need to measure anxiety and depression using the patient portal? When should these assessments be sent out? Before scheduled clinic visits? Proactively even if patients don’t have scheduled visits? How frequently for screening? How frequently for patients with anxiety and depression?
5. Do you think assessing anxiety and depression using the patient portal will be effective? Why or why not?
6. What are potential barriers to implementing measuring anxiety and depression using the patient portal?
7. How will assessing anxiety/depression using the portal impact your work?
8. What are ways to make the portal impact your work as least as possible?
9. What do you think should happen to the information collected from the patient portal about anxiety/depression symptoms?
10. Who should be responsible for addressing to the information collected from the patient portal? For positive results? For negative results? How would you prefer to be alerted about a patient’s anxiety and depressive positive screening results? What about for negative results?
11. How do you think the program needs to be customized for PLWH? For PLWH with different levels of anxiety and depression severity?
12. Do you think measuring anxiety and depression using the patient portal will meet the needs of the patients served by your clinic? Why or why not?
13. The following individuals are involved in this project: __. Who are other key influential individuals to get on board with assessing anxiety and depressive symptoms using the portal?

Other possible strategies

1. Do you think your patients would feel comfortable answering question about anxiety and depression symptoms by text or on tablets or their phones in the waiting area? Why or why not?
2. How to leverage current efforts/resources in HIV clinic to improve anxiety and depression screening and management?
3. What services or resources are your patients getting in the community pertaining to mental health care? What services do patients in the clinic need that they aren’t getting?
4. What types of interventions would you like to see in this clinic?
5. What else do you think we should be asking your patients about to help with their healthcare?

Wrap up

1. Is there anything else that you would like to share that I did not ask about?
